# Antimicrobial resistance patterns of *Escherichia coli* detected from milk, milking environment, and milk consumers in Addis Ababa milksheds, Ethiopia

**DOI:** 10.3389/fvets.2026.1984452

**Published:** 2026-10-01

**Authors:** Sitota Tesfaye Tekle, Madelaine Norström, Gro Skøien Johannessen, Jannice Schau Slettemeås, Fasil Ejigu Eregno, Gezahegne Mamo

**Affiliations:** 1 Department of Veterinary Epidemiology and Public Health, College of Veterinary Medicine and Agriculture, Addis Ababa University, Bishoftu, Ethiopia; 2 Department of Building, Energy and Material Technology, Faculty of Engineering Science and Technology, UiT The Arctic University of Norway, Tromsø, Norway; 3 Norwegian Veterinary Institute, Ås, Norway

**Keywords:** Addis Ababa, antimicrobial resistance, *Escherichia coli*, milk, milk consumers, milking environment

## Abstract

Antimicrobial resistance (AMR), primarily caused by irrational antimicrobial use in humans, animals, and agriculture, poses a serious global public health threat, particularly in resource-limited low- and middle-income countries, where milk processing, hygiene standards, and prudent antimicrobial use regulations are minimal. Previous studies in Ethiopia reported the prevalence of AMR in *Escherichia coli* bacteria in milk and dairy environments but have overlooked the relationships to milk consumers. This study aimed to investigate AMR patterns in *E. coli* isolated from milk, the milking environment, and milk consumers in Addis Ababa milksheds in Ethiopia. A cross-sectional study design was used to collect 404 samples from proportional simple randomly selected milksheds in Addis Ababa, from which raw bulk milk, stool from milk consumers, and milking-environment samples were collected. *E. coli* was isolated and identified using standard bacteriological protocols and confirmed by Matrix-Assisted Laser Desorption/Ionization Time-of-Flight Mass Spectrometry (MALDI-TOF MS). Isolates were tested for antimicrobial susceptibility against twelve antimicrobials from eight antimicrobial classes using the Kirby-Bauer disk diffusion method on Mueller-Hinton agar, following the guidelines of the European Committee on Antimicrobial Susceptibility Testing (EUCAST). Multidrug resistance (MDR) and multiple antimicrobial resistance indices (MARI) were calculated, and analyses were performed using R. Presumptive *E. coli* was isolated from 18.6% and confirmed in 12.6% of the 404 samples analyzed. Overall, 84.3% of isolates were resistant to at least one antimicrobial tested, with the highest resistance rates for gentamicin (64.7%), ampicillin (52.9%), and tetracycline (41.2%), while resistance to ceftazidime (5.9%) and cefotaxime (7.8%) were low. MDR was observed in 50.9% of the isolates, with MARI values ranging from 0.17 to 1. The study revealed a low detection rate of *E. coli* but a considerably high proportion of AMR in Addis Ababa milksheds, Ethiopia. The finding underscores the need for targeted One Health surveillance to curb the emergence and simultaneous occurrences of antimicrobial-resistant *E. coli* at dairy production interfaces and safeguard public health.

## 1 Background

Antimicrobials are used in public and animal health settings to reduce mortality and morbidity in humans and animals. Antimicrobial resistance (AMR), resulting from the irrational use of antimicrobials in public health care, animal health care, and agriculture, accelerates the emergency of antimicrobial resistance pathogens and poses a serious threat to public health (1). AMR is rapidly spreading around the world, irrespective of political, geographic, or economic differences, and it has an enormous impact on low- and middle-income countries (LMICs). It occurs when bacteria, viruses, fungi, and parasites evolve to become resistant to antimicrobials, making infections difficult to treat and increasing the risk of disease transmission, severity, and death (2).

AMR increases production costs, reduces animal health, and raises public health costs due to the need for expensive last-generation antimicrobials and longer hospitalizations (3). A recent estimate indicated 1.27 million bacterial AMR-attributable deaths worldwide (2). It was projected that AMR could cost more than $1 trillion (4) and could result in 10 million deaths and an 11% annual decline in global livestock productivity by 2050 if no effective action is taken (5).

The primary driver of the emergence and spread of AMR in Gram-negative normal microbiota is selective pressure from the extensive use of antimicrobials in apparently healthy food-producing animals (6, 7). *E. coli* is a member of the Gram-negative normal microbiota of the human and animal gastrointestinal tracts; however, some strains have become pathogenic due to acquired virulence factors and AMR genes (8). *E. coli* is frequently used as indicator and sentinel bacteria to assess the patterns and profiles of AMR co-occurrence in One Health settings, as they are widely distributed bacteria and can easily acquire and disseminate antimicrobial resistance (9). *E. coli* is a high-priority pathogen of public health concern due to its resistance to third-generation cephalosporins and carbapenems and the emergence of multidrug resistance (MDR) linked to a variety of factors, including mobile genetic elements such as plasmids, transposons, and bacteriophages that can be exchanged between bacteria and confer resistance to a variety of antimicrobials (8, 10).

Despite Ethiopia’s inclusion in the global initiative against the threat of AMR through a national strategic plan and a multisectoral framework following the One Health approach, Ethiopia’s AMR response is fluctuating due to resource constraints, fragmented sectorial participation, and weak policy implementation at the local level (11, 12). Ethiopia, like other LMICs, has unrestricted access to antimicrobials; suboptimal hygiene standards, combined with weak regulatory controls; limited effective surveillance; and insufficient diagnostic and laboratory capacity, all of which contribute to uncontrolled and inappropriate use of antimicrobials in food-producing animals, including dairy cattle (12, 13). Due to extensive informal milk marketing channels coupled with limited processing of milk in Ethiopia, milk might be contaminated with *E. coli* that may function as a hotspot for co-occurrence of antimicrobial-resistant *E. coli* bacteria at the dairy production and consumption interfaces (14).

Previous studies in Ethiopia have documented the prevalence of AMR in milk and dairy farm environments in *E. coli* (9, 10, 15, 16), with limited investigation into how AMR patterns in milk and dairy farm environments influence AMR patterns among milk consumers. Therefore, this study aimed to fill this important gap by investigating the AMR patterns in *E. coli* in milk, the milking environment, and milk consumers in Addis Ababa milksheds, through understanding the interconnected nature of human, animal, and environmental health in the One Health concept in Ethiopia. Accordingly, we hypothesized that AMR patterns in *E. coli* from milk and the milking environment would be reflected in *E. coli* isolated from milk consumers, indicating co-occurrence of antimicrobial-resistant bacteria at the human-animal-environment interface.

## 2 Materials and methods

### 2.1 Study areas

This study was conducted in three purposively selected sub-cities of Addis Ababa, the capital of Ethiopia, based on the high concentration of urban dairy farms and dense populations: Akaki Kality, Nifas Silk Lafto, and Kolfe Karanio (Figure 1). Addis Ababa is located at 9°1′48″N latitude and 38°44′24″E longitude, with an average elevation of 2,355 meters above sea level. The city’s weather changes seasonally, with the rainy season lasting from June to September and the dry season lasting from October to May. The average annual temperature is about 21 °C, with annual rainfall averaging 1,800 mm. Addis Ababa covers 527 square kilometers and is divided into 11 administrative sub-cities with a population of approximately 4.8 million (17).

**Figure 1:**
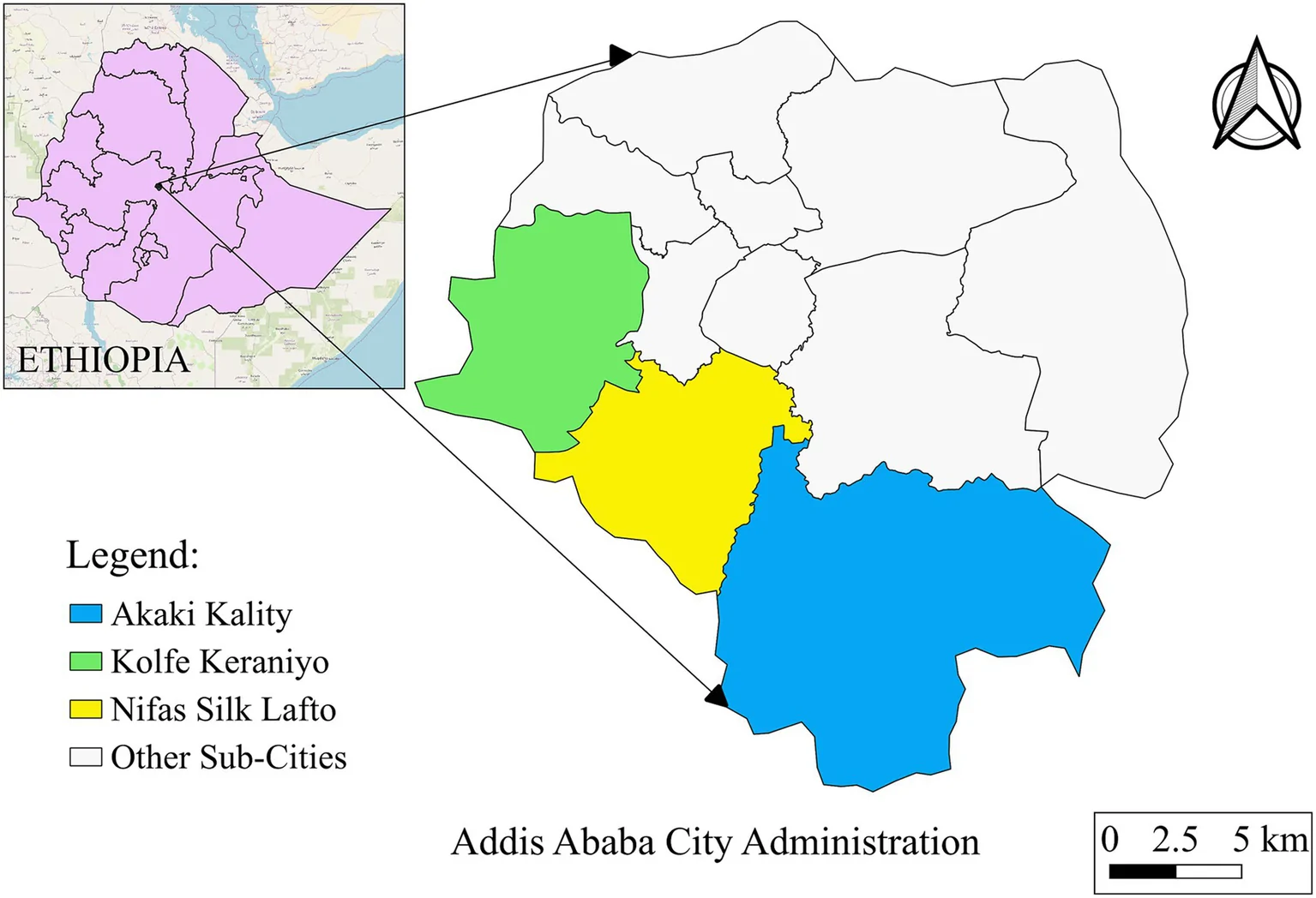
Map of the study areas generated by QGIS.

### 2.2 Study settings

Samples were collected from pooled bulk milk, the milking environment, and the stool of milk consumers. Milk samples were collected from dairy farms where dairy cattle supplied milk to one or more consumers or acted as retailers. The dairy cattle at the study site were mainly pure Holstein Friesian and crossbreeds, and they were intensively managed for milk production. Swabs from the floor and the water used for washing teats, hands, or equipment as well as animals’ consumption were collected as environmental samples. Human stool samples were collected from apparently healthy individuals who have a history of consuming raw milk or milk products from the corresponding included milksheds (18).

### 2.3 Study design and sample size determination

A cross-sectional study design was used to collect all samples from April 2025 to January 2026. The sample size for milk was calculated using the formula outlined by Thrusfield (19), with a 95% confidence level, an absolute precision of 5%, and an expected prevalence of 7.1% for *E. coli* in central Ethiopia (16). Based on this calculation, 101 bulk milk samples were collected using a proportional simple random sampling method. A total of 101 milk sheds were included in this study, and one bulk milk, floor swab, water, and human stool sample per included milkshed were collected. A total of 202 environmental samples were collected along with the bulk milk samples. A total of 101 human stool samples were included from people with a history of drinking milk from the milksheds. The sample size was determined using Thrusfield’s formula (19), with an expected prevalence of 2.8% (20), a 95% confidence level, and 5% precision, which generated a sample size of 42. To improve the precision and reliability of prevalence estimates in the apparently healthy human population, the sample size increased to 101. Stool samples were collected from randomly selected individuals who had a history of consuming milk from the corresponding included milksheds. The inclusion criteria for human stool sample collection of both sexes were defined as the history of drinking milk from the included milkshed, apparent health conditions of the individuals, and age greater than 18 for the consent purpose. Accordingly, individuals who had no history of drinking milk, were diseased, and were below 18 were excluded during human stool sample collection. Accordingly, this study collected and analyzed 404 samples, which included 101 bulk tank milk samples, 101 farm floor surface swabs, 101 water samples, and 101 human stool samples.

### 2.4 Sample collection strategy

#### 2.4.1 Milk sample collection

After thoroughly shaking the buckets that contained milk from all cows in the milksheds, approximately 50 mL of milk sample was collected from pooled bulk tank milk into sterile screw-cap tubes (13).

#### 2.4.2 Environmental sample collection

The floor swab samples were collected from the milking facility using a sterile cotton-tipped swabbing technique, in accordance with standard protocols for microbial surface sampling and human biological trace collection (21). The swab tip was moved across the surface in a zigzag pattern, like a wavy line moving back and forth. The swab was then swept perpendicularly across the surface a second time in the same direction as in the earlier sweep, and it was immediately placed in a sterile screw-cap tube containing 10 mL of Buffered Peptone Water (BPW) (Oxoid, UK). The swab samples from the floor surface were collected immediately prior to milking. Water (50 mL) used by farmers to wash teats, hands, or equipment and drunk by their animals was collected aseptically into sterile screw-capped universal tubes (18).

#### 2.4.3 Human stool sample collection

Medical laboratory personnel collected human stool samples in sterile, disposable screw-cap tubes labeled with the appropriate identification. Approximately 1 gram of freshly passed stool was transferred into tubes containing 10 mL of BPW (22). All 404 samples were labeled with waterproof ink and delivered to the bacteriological section of the Wolaita Sodo Regional Veterinary Laboratory in an icebox filled with ice packs. All samples were stored at 4 °C until testing and analyzed within 24–48 h of collection (9).

### 2.5 Isolation and identification of *Escherichia coli*

Isolation of *E. coli* was performed following the guidelines of Quinn et al. (23), with minor modifications. After thorough mixing, 25 mL of raw milk and 25 mL of water samples were transferred to separate stomacher bags containing 225 mL (1:9) of BPW. The homogenate was vortexed and incubated at 37 °C for 18–24 h. Floor surface swab and stool samples (1 gram), which were homogenized in BPW, were immediately incubated at 37 °C for 18–24 h upon arrival at the laboratory. After enrichment, a loopful of each homogenate was streaked onto MacConkey agar (Oxoid, UK) and incubated at 37 °C for 18–24 h. Growth of *E. coli* was identified by round pink-to-red colonies with precipitation on MacConkey agar. Two to three presumptive colonies of *E. coli* per sample were recovered by re-streaking on an Eosin Methylene Blue agar (EMBA) (Oxoid, UK), which was further incubated at 37 °C for 18–24 h. Colonies showing a green metallic sheen on the EMBA were then inoculated onto nutrient agar (Oxoid, UK) to ensure purity. Biochemical tests, i.e., Triple Sugar Iron (TSI), indole production, citrate reduction, urease production, methyl red, and Voges-Proskauer, were performed to confirm presumptive *E. coli* (23).

Further laboratory confirmatory analysis was conducted at the Norwegian Veterinary Institute. One randomly selected presumptive *E. coli* isolate per each sample was confirmed using MALDI-TOF MS (Bruker MALDI Biotyper, Bremen, Germany) following the manufacturer’s instructions. The results with log scores of 2 or higher were used to confirm species (24).

### 2.6 Antimicrobial susceptibility testing

Antimicrobial susceptibility testing of the *E. coli* isolates was performed using the standardized Kirby-Bauer disk diffusion method on Mueller-Hinton agar (Oxoid, UK) in accordance with the EUCAST guidelines (25). The testing included twelve antimicrobials from eight different antimicrobial classes. The availability, use in public and veterinary healthcare facilities, and feed additives to promote animal growth were determining factors in the choice of antimicrobials (18, 26). Each *E. coli* isolate was suspended in 5 mL of sterile saline, and the turbidity was set to match a 0.5 McFarland standard. The suspension was spread onto Mueller-Hinton agar plates at a volume of 0.1 mL. Antimicrobial disks were placed on the plates and incubated at 37 °C for 18–24 h. After incubation, inhibition zones were measured using a caliper, and results were interpreted. EUCAST clinical breakpoints version 16.1-based categorizing of isolates were made as susceptible (S), susceptible increased exposure (I), and resistant (R) (25). Isolates categorized as I categories were grouped together with susceptible isolates before further analysis in our study. For antimicrobial agents lacking established clinical breakpoints for *E. coli*, zone diameters were interpreted using EUCAST epidemiological cut-off values (ECOFFs). ECOFFs classify isolates as wild-type (WT) and non-wild-type (NWT). For neomycin and tetracycline antimicrobials lacking clinical breakpoints and ECOFFs for *E. coli*, VetCAST criteria were used to categorize isolates as wild-type and non-wild-type. The ECOFFs cutoff values classify bacterial populations lacking acquired/mutational resistance mechanisms as wild type (WT) and those with phenotypically expressible resistance mechanisms as non-wild type (NWT) (Table 1) (27, 28). The *E. coli* (ATCC 25922) reference strain was used for quality control during culturing and antimicrobial susceptibility testing.

**Table 1:** Tested antimicrobials and their EUCAST clinical cutoff values for interpretation.

Class	Antimicrobial	Potency (μg)	S ≥ (mm)	R < (mm)	I (mm)	Interpretation
Aminoglycosides	Neomycin	30	–	13*	–	VetCAST ECOFF
	Gentamicin	10	17	17	–	
Amphenicols	Chloramphenicol	30	–	17	–	EUCAST Screening
Cephalosporins	Ceftazidime	10	22	19	19–21	
	Cefotaxime	5	20	17	17–19	
Nitrofurans	Nitrofurantoin	100	–	13*	–	ECOFF
Penicillins	Ampicillin	10	14	14	–	
Quinolones	Ciprofloxacin	5	25	22	22–24	
	Nalidixic acid	30	–	19*	–	ECOFF
Antifolates	Trimethoprim/sulfamethoxazole	25	15	15	–	
	Trimethoprim	5	–	20*	–	ECOFF
Tetracyclines	Tetracycline	30	–	19*	–	VetCAST ECOFF

*Unless otherwise stated, zone diameters are interpreted using EUCAST clinical breakpoints. For the ease of reading the table, ECOFFs are interpreted as susceptible and resistant instead of wild type and non-wild-type, respectively. ECOFF, EUCAST epidemiological cutoff values.

Multidrug-resistant isolates, defined as those isolates resistant to at least one antimicrobial in three or more antimicrobial classes (29), were identified. The MARI was calculated using Krumperman’s (1983) formula (30), MARI = a/b, where ‘a’ indicates the number of antimicrobials the bacterial isolate is resistant to and ‘b’ indicates the total number of antimicrobials tested against that isolate. For the purposes of statistical analyses, MDR determination, and MARI calculations in this study, WT and NWT classifications were recoded as susceptible and resistant, respectively. A MARI index of 0.20 or higher was compatible with exposure to environments under substantial antimicrobial pressure (9, 31).

### 2.7 Data management and analysis

The data were entered, edited, coded, and stored using Microsoft Office 365, and statistical analysis was conducted in R version 4.5.2 (R Core Team, 2025). The results were summarized with descriptive statistics and shown in tables and figures. A repeated-measures binary approach, such as a Cochran’s Q test with milkshed as the clustering unit, was used to determine the association between *E. coli* positivity and explanatory variables. Resistance proportions were reported against each antimicrobial tested. Statistical significance was determined using 95% confidence intervals, and *p* values ≤ 0.05 were considered significant. MARI was analyzed as a numerical variable, and distribution patterns were observed using box plots. The Kruskal-Wallis *p*-value was used to observe differences within groups.

## 3 Results

### 3.1 Occurrence of antimicrobial resistance in *Escherichia coli*

Presumptive *E. coli* was detected by culture and biochemical identification in 75 (18.6%) of 404 samples collected and confirmed by MALDI-TOF MS in 51 (12.6%). The detection rate of *E. coli* significantly varies across sample sources and sites (*p*-value < 0.05) (Table 2). The overview of the samples collected and analyzed by sample sources and site was summarized (Table 3). Overall, *E. coli* isolates exhibited higher phenotypic resistance to gentamicin (64.7%), ampicillin (52.9%), and tetracycline (41.2%). Conversely, relatively low resistance rates were observed for ceftazidime (5.9%) and cefotaxime (7.8%) (Figure 2). Resistance profiles of isolates varied by sample source and sampling site. Akaki Kality and Nifas Silk Lafto exhibited higher levels of gentamicin resistance (70 and 64.3%, respectively) (Figure 3). Among sample sources, isolates of human stool were found to be more resistant to gentamicin (70%), nalidixic acid (45%), and tetracycline (45%). Isolates from milk were resistant to gentamicin, ampicillin, trimethoprim, and tetracycline at rates of 61.9, 57.1, 43, and 33.3% resistance, respectively. Water isolates from the environmental compartment showed high resistance to gentamicin (75%), and floor isolates showed high resistance to ampicillin (66.7%), trimethoprim (66.7%), and trimethoprim/sulfamethoxazole (66.7%) (Figure 4).

**Table 2:** Detection rates of *Escherichia coli* in the study settings.

Variable	Sample sources	No. of samples	No. of positive	Proportion % (95% CI)	*Q*	*p*-value
Source	Bulk milk	101	21	20.8 (13.4–30)	22.94	< 0.001
	Human Stool	101	20	19.8 (12.5–28.9)		
	Floor	101	6	5.9 (2.21–12.5)		
	Water	101	4	3.9 (1.09–9.83)		
Site	Akaki Kality	216	20	9.3 (5.8–13.9)	9.5	0.023
	Nifas Silk Lafto	104	14	13.5 (7.6–21.6)	12.9	0.004
	Kolfe Karanio	84	17	20.2 (12.3–30.4)	10.2	0.017
Overall		404	51	12.6 (9.5–16.3)		

CI, confidence interval.

**Table 3:** Overview of samples collected, distributed by sample site and source.

Sample site	Human stool	Floor	Water	Bulk milk	Total
Akaki Kality	54	54	54	54	216
Nifas Silk Lafto	26	26	26	26	104
Kolfe Karanio	21	21	21	21	84
Total	101	101	101	101	404

**Figure 2:**
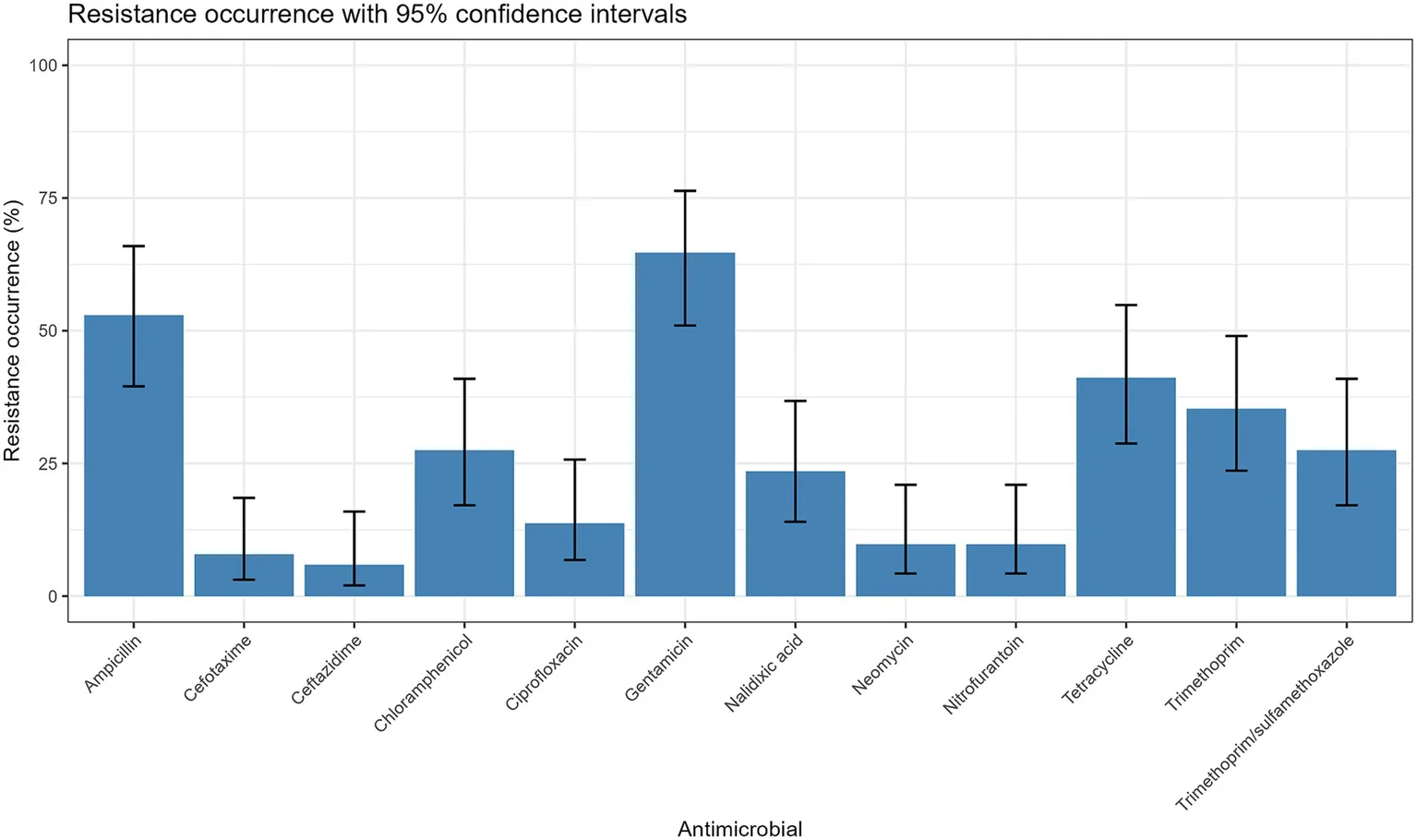
Prevalence of antimicrobial resistance in *Escherichia coli* from all sample sources against selected antimicrobials.

**Figure 3:**
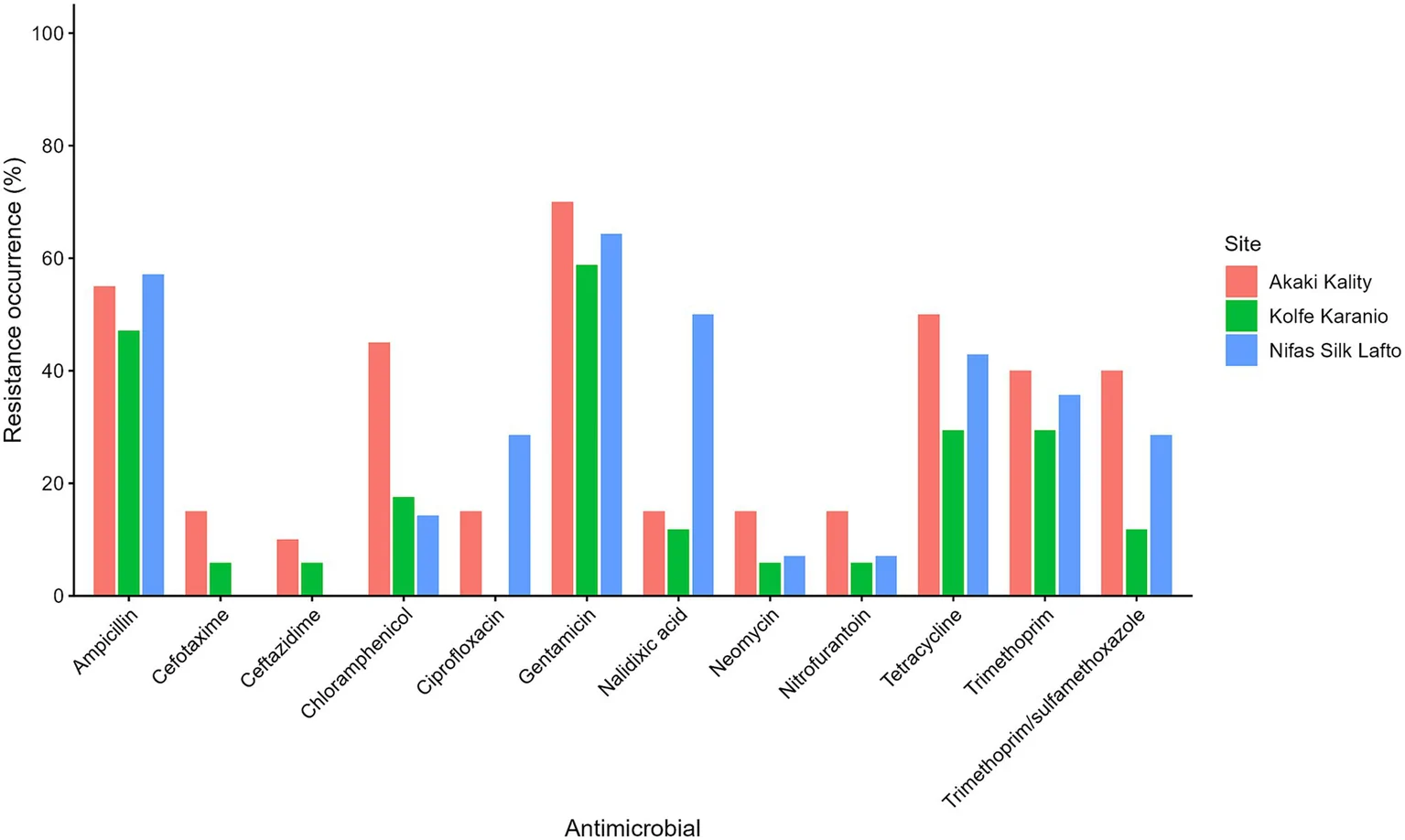
Occurrence of antimicrobial resistance in *Escherichia coli* varied by site.

**Figure 4:**
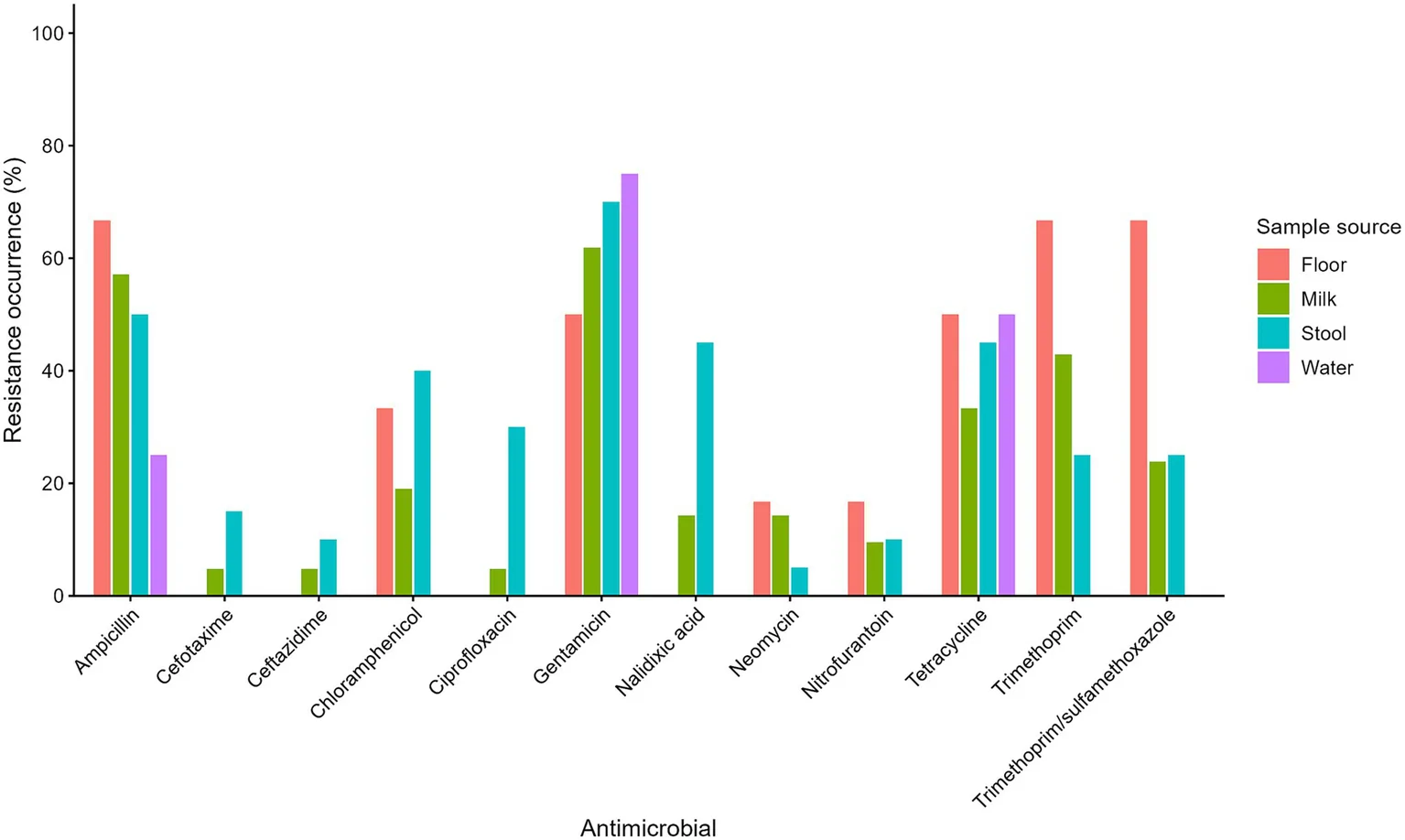
Occurrence of antimicrobial resistance in *Escherichia coli* varied by sample source.

### 3.2 Occurrence of MDR and MAR index

Overall, 8 (15.7%) *E. coli* isolates were completely susceptible to all antimicrobials tested, while 43/51 (84.3%) were resistant to at least one antimicrobial tested, and 42/51 (82.4%) isolates were resistant to at least two classes of antimicrobials tested, whereas a small percentage of isolates (2%) demonstrated resistance to all antimicrobial classes tested (Table 4). MDR was observed in 26/51 (50.9%) of *E. coli* isolates, with MAR index values ranging from 0.17 to 1. The predominant MDR profiles were found for antimicrobial combinations involving AMP, SXT, TMP, CN, C (11.5%); AMP, SXT, TE, TMP (7.7%); and AMP, TMP, CN (7.7%) (AMP-Ampicillin; SXT-Sulfamethoxazole-trimethoprim; TMP-Trimethoprim; CN-Gentamicin; C-Chloramphenicol; TE-Tetracycline) (Table 5). No significant variation in MDR prevalence was observed in the sample sources (*p* = 0.21) and in the sampling site (*p* = 0.52) (Table 6). MARI did not show a significant difference between the sample sources (Figure 5) and the site (Figure 6) (Kruskal–Wallis test, *p* > 0.05). MARI inconsistencies were observed in human stool, Akaki Kality, and Nifas Silk Lafto.

**Table 4:** Distribution of the resistance pattern of isolates to antimicrobial classes.

Antimicrobial classes	Number of isolates	Percent
Resistance to none	8	15.7
Resistance to one	1	2
Resistance to two	16	31.4
Resistance to three	13	25.5
Resistance to four	8	15.7
Resistance to five	2	3.9
Resistance to six	1	2
Resistance to seven	1	2
Resistance to eight	1	2

**Table 5:** MDR index and MDR status of *Escherichia coli* isolates.

AMR isolate	AMR profile	Classes of AM	Number of AM-resistant	MDR index	MDR status
ak102	AMP, SXT, TE, NA, CIP, N, TMP, CTX, CAZ, CN, C, F	8	12	1	MDR
S2	AMP, SXT, TE, TMP, CTX, CN, C, F	7	8	0.67	MDR
S25	AMP, TE, NA, CTX, CAZ, CN, C	6	7	0.58	MDR
S3	AMP, TE, CTX, CAZ, CN, C	5	6	0.5	MDR
ak120	AMP, SXT, TMP, CN, C, F	5	6	0.5	MDR
s21	AMP, SXT, NA, CIP, TMP, F	4	6	0.5	MDR
ak76	AMP, SXT, TMP, CN, C	4	5	0.42	MDR
ak79	AMP, SXT, TMP, CN, C	4	5	0.42	MDR
kk80	AMP, SXT, TE, TMP, CN	4	5	0.42	MDR
s48	AMP, SXT, TMP, CN, C	4	5	0.42	MDR
aK18	AMP, TE, N, CN	3	4	0.33	MDR
N6	AMP, N, TMP, CN	3	4	0.33	MDR
O3	SXT, N, TMP, C	3	4	0.33	MDR
O7	AMP, NA, TMP, F	4	4	0.33	MDR
S14	NA, CIP, N, CN	2	4	0.33	Non-MDR
S15	NA, CIP, CN, C	3	4	0.33	MDR
S9	AMP, NA, CIP, CN	3	4	0.33	MDR
ak101	SXT, TE, TMP, CN	3	4	0.33	MDR
ak38	AMP, SXT, TE, TMP	3	4	0.33	MDR
kk57	AMP, NA, CN, C	4	4	0.33	MDR
kk60	AMP, TE, CN, C	4	4	0.33	MDR
kk70	AMP, SXT, TE, TMP	3	4	0.33	MDR
N2	AMP, TE, CN	3	3	0.25	MDR
O2	AMP, TMP, CN	3	3	0.25	MDR
O4	AMP, TMP, CN	3	3	0.25	MDR
S11	NA, CIP, CN	2	3	0.25	Non-MDR
S19	AMP, TE, C	3	3	0.25	MDR
S8	SXT, NA, TMP	2	3	0.25	Non-MDR
ak81	SXT, TE, TMP	2	3	0.25	Non-MDR
s33	TE, CN, C	3	3	0.25	MDR
M2	TE, CN	2	2	0.17	Non-MDR
M4	AMP, CN	2	2	0.17	Non-MDR
N1	TE, CN	2	2	0.17	Non-MDR
N5	TE, CN	2	2	0.17	Non-MDR
S10	NA, CIP	1	2	0.17	Non-MDR
S12	NA, CN	2	2	0.17	Non-MDR
S13	AMP, TE	2	2	0.17	Non-MDR
S28	TE, CN	2	2	0.17	Non-MDR
S4	AMP, CN	2	2	0.17	Non-MDR
ak100	AMP, CN	2	2	0.17	Non-MDR
ak87	TE, CN	2	2	0.17	Non-MDR
kk55	TE, CN	2	2	0.17	Non-MDR
kk79	AMP, CN	2	2	0.17	Non-MDR

AMR, Antimicrobial resistance; AM, Antimicrobials; MDR, Multidrug resistance; AMP, Ampicillin; SXT, Sulfamethoxazole-trimethoprim; TE, Tetracycline; NA, Nalidixic acid; CIP, Ciprofloxacin; N, Neomycin; TMP, Trimethoprim; CTX, Cefotaxime; CAZ, Ceftazidime; CN, Gentamicin; C, Chloramphenicol; F, Nitrofurantoin.

**Table 6:** Distribution of MDR patterns stratified by sample source and site.

Variables	Isolates source	Positive isolates	MDR *n* (%)	*χ* ^2^	*p*-value
Sample source	Bulk milk	21	11 (52.4)	4.89	0.21
	Human stool	20	11 (55)		
	Floor	6	4 (66.7)		
	Water	4	0		
Site	Akaki Kality	20	12 (60)	1.31	0.52
	Nifas Silk Lafto	14	7 (50)		
	Kolfe Karanio	17	7 (41.2)		

**Figure 5:**
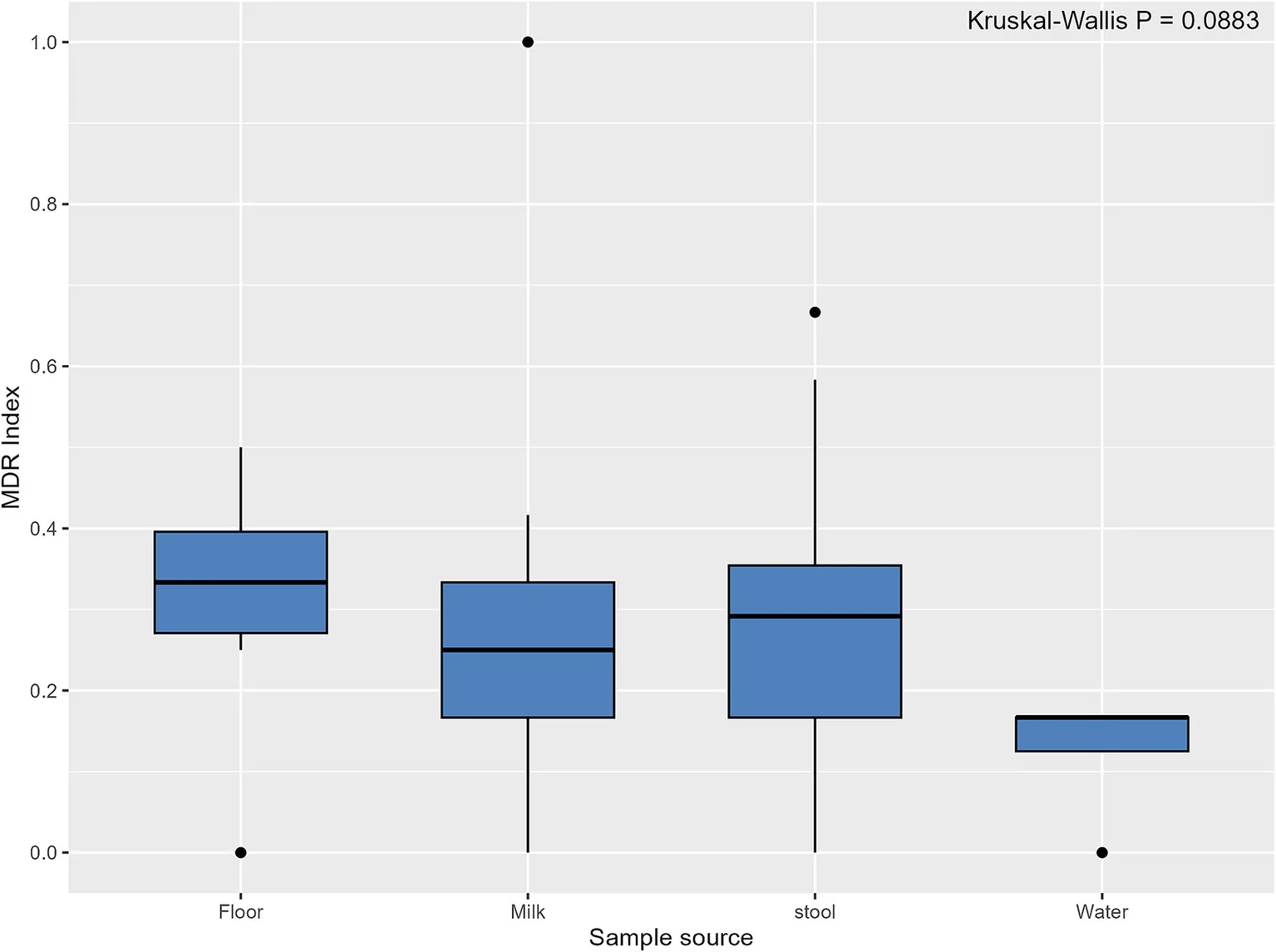
Multidrug resistance index of *Escherichia coli* by sample source.

**Figure 6:**
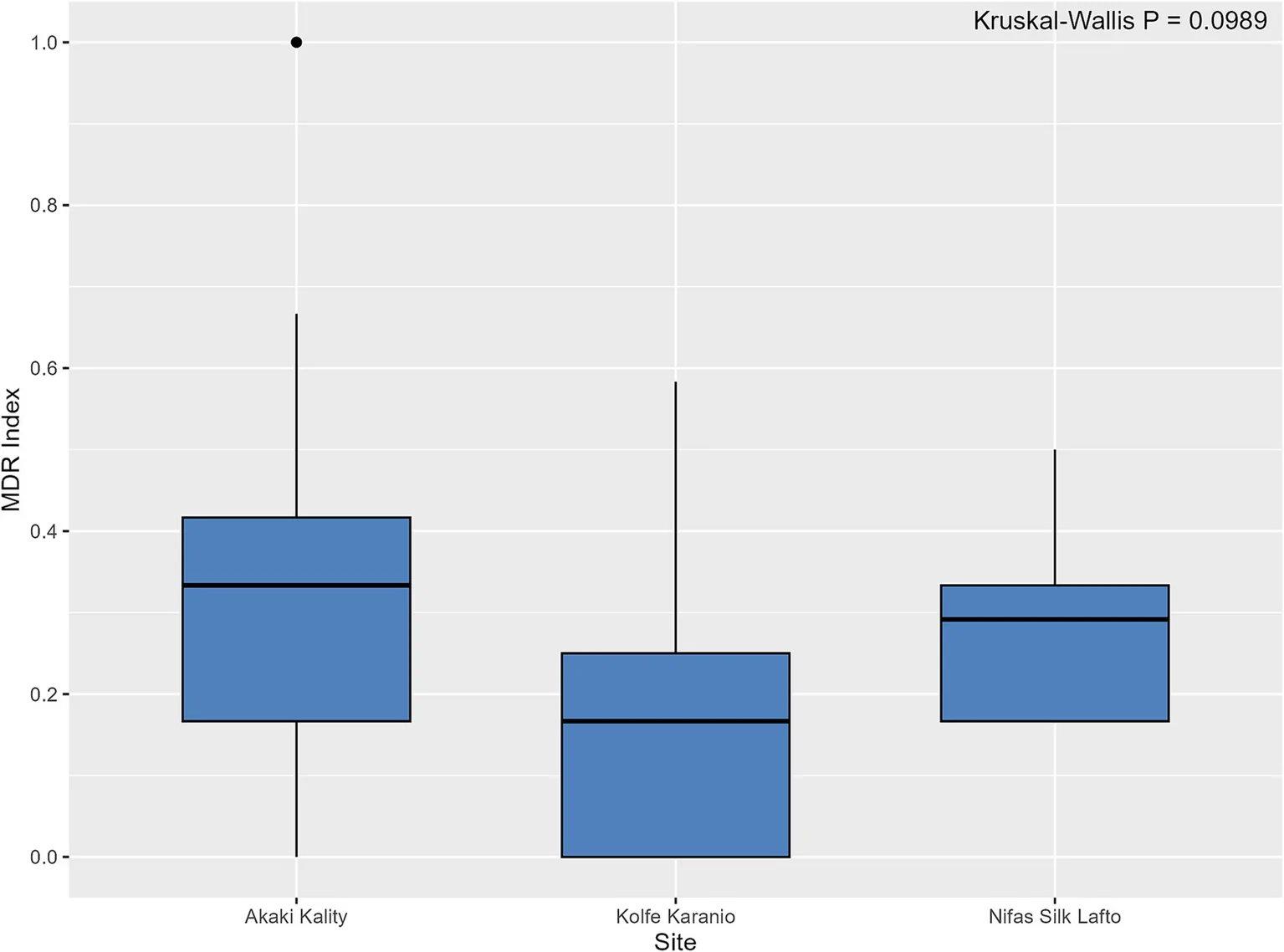
Multidrug resistance index of *Escherichia coli* by site.

## 4 Discussion

This study detected antimicrobial-resistant *E. coli* in milk and the milking environment and among milk consumers in Addis Ababa, Ethiopia. This simultaneous detection of antimicrobial-resistant *E. coli* at both the dairy production and consumption interfaces is a cause for public health concern (13). Despite being normal microbiota in the gastrointestinal system, *E. coli* may trigger mastitis and contribute to treatment failures in dairy animal farming (9). Humans may be exposed to antimicrobial-resistant *E. coli* bacteria through the food chain or direct contact with animals that results in increased risk of dissemination of antimicrobial resistance and complicates the treatment of infections, making treatment more challenging since isolates may carry resistance to critically important or last-resort antimicrobials (8). As a result, our findings can be used to inform the development of an integrated One Health framework to guide the focused intervention strategy on AMR stewardship in resource-limited LMICs like Ethiopia.

In our study, the overall detection rate for *E. coli* was 12.6%; in contrast, a higher overall prevalence of *E. coli* (50.9%) was reported from Ethiopia (10). Our study revealed that 20.8% of milk samples were contaminated with *E. coli*. Similarly, other studies have reported diverse contamination rates from 7.1 to 81.99% in several countries, including Ethiopia (8, 13, 16, 31–34). This discrepancy in the prevalence of *E. coli* in various locations might be attributed to differences in geographic location, variation in sample size, study population, isolation techniques, sampling, and milking techniques, as well as hygiene practices (8, 13).

In this study, the occurrence of *E. coli* in stool samples from apparently healthy humans who have a history of drinking milk was 19.8%; in contrast, a higher prevalence rate was reported in milkers’ stool (75.6%) (10) and in contact human stool, 67% (35), in Ethiopia. Studies in other countries also reported a high prevalence of *E. coli* in attendants’ human stool from Tanzania (36) and fecal material from Kenya (37). The observed low detection rate of *E. coli* in our study in the stool of apparently healthy adult humans might be associated with alterations in the composition of adult gut microbiota linked to recent antimicrobial use, the presence of heavy metals, differences in diet, travel history, and other disease conditions (38) or it could be due to methodological limitations. In our study, 5.9 and 3.9% of floor and water samples of the environmental domain were tested positive for *E. coli*. In contrast, another study (39) reported a relatively high prevalence from environmental-origin samples.

Our findings revealed that 84.3% of *E. coli* isolates were resistant to at least one antimicrobial tested, which aligns with reports from Ethiopia that indicated high levels of resistance to one or more antimicrobials (9, 13). Other studies from different countries also reported comparable findings (8, 40, 41). The present study revealed high resistance to aminoglycosides (gentamicin, 64.7%), beta-lactams (ampicillin, 52.9%), and tetracyclines (41.2%). This finding is consistent with other reports of tetracycline and aminoglycoside resistance from Kenya (37) and is comparably lower than the report for tetracycline (72%) from Ethiopia (10). This difference in the prevalence of resistance to aminoglycosides, beta-lactams, and tetracyclines observed in different studies might be linked to the difference in antimicrobial use for therapeutic and prophylactic purposes in human and veterinary medicine (8, 9). We observed relatively low rates of resistance to cephalosporins such as ceftazidime (5.9%) and cefotaxime (7.8%). These detection proportions were comparable to previous reports from central and northern Ethiopia for ceftazidime (10, 13) but comparably lower than the 19.8% resistance for cefotaxime from northern Ethiopia (10). This difference in the prevalence of resistance to third-generation cephalosporins in various studies might be linked to the difference in the use of these antimicrobials in veterinary and human medicine (42).

In this study, *E. coli* isolates from the milk source were found resistant to gentamicin (62%), nitrofurantoin (9%), nalidixic acid (14%), cefotaxime (4.7%), ceftazidime (4.7%), and ciprofloxacin (4.7%). In agreement with the current study, relatively comparable resistance to cefotaxime, ceftazidime, and ciprofloxacin and relatively lower resistance to nalidixic acid were reported from Egypt (43). In contrast, a relatively high prevalence of resistance to nitrofurantoin (23%), nalidixic acid (24%), and ciprofloxacin (24%) and a lower gentamicin resistance (16%) were reported previously in Ethiopia (9). Another study reported a distinct finding of complete absence of resistance in all isolates to nalidixic acid and ciprofloxacin in Kenya (8). Previous research suggests that the difference in the resistance proportion for antimicrobials rarely used in the treatment of dairy animals might be attributed to the transmission of resistance determinants such as extended-spectrum beta-lactamase (ESBL) genes, indirect exposure from human cephalosporin use leading to environmental contamination, direct contact between livestock and humans, or co-selection caused by extensive use of oxytetracycline, penstrep, and sulfonamide in animals. Such resistance poses a risk that expands beyond increased treatment failure in animals and likely spreads to humans through animal-derived foods and environmental circulation (9).

In our study, MDR was observed in 50.9% of *E. coli* isolates, which is relatively comparable to reports from Ethiopia (9). Other studies reported relatively higher MDR rates in Ethiopia (16, 35, 44) and Bangladesh (31); conversely, lower MDR rates were reported in Kenya (8) and Zambia (45). The discrepancy could be attributed to the difference in national action plans on AMR, an improved understanding of antimicrobial use and resistance, and monitoring and controlling the spread of antimicrobial-resistant bacteria to the environment, animals, and humans (10, 46). This study detected the MAR index value ranging from 0.17 to 1 in *E. coli* isolates. A previous study in Ethiopia reported relatively comparable MARI values between 0.3 and 0.9 from milk isolates of *E. coli* (9). A relatively lower MARI value was reported from China (47). This high level of MARI value in our study underscores compatibility with exposure to environments under substantial antimicrobial pressure that can be linked to poor antimicrobial use control regulations implementation in the current study area (48).

To the best of our knowledge, this was the first study that linked milk and milk consumers to investigate the AMR in *E. coli* associated with milk, milking environment, and milk consumer in Addis Ababa, Ethiopia. Ethiopia, as an LMIC with a lack of food safety regulations and standards, limited diagnostic capabilities at national and regional levels (49), and a general preference of consumers for unprocessed milk due to a variety of reasons (50), consumers might be directly exposed to antimicrobial-resistant bacteria without disease exposure. Our findings of high prevalences of AMR, MDR, and MARI in milk, the milking environment, and milk consumers’ stool underscore the national threat of AMR bacteria and align with the World Health Organization’s Global Action Plan on AMR and the One Health framework, which emphasize the interconnectedness of human, animal, and environmental health (51). MDR pathogens are a major global health threat, as they cause infections with few or no effective treatment options, leading to increased morbidity and mortality, with the highest burden in sub-Saharan Africa, including Ethiopia (2, 31). They also raise healthcare costs and complicate infection control in hospitals, communities, and veterinary healthcare settings (3). Therefore, detecting indicator bacteria like *E. coli* at human-animal-environmental interfaces in the dairy production system allows for targeted intervention in hygiene practices, antimicrobial use, and surveillance, ensuring safe milk production and consumption at various levels and nationwide.

## 5 Study limitations

The limitations of this study were its extent, which is limited to the Addis Ababa metropolitan region of Ethiopia; the study design and the relatively small sample size collected due to resource constraints, which may have a negative impact on the generalizability of the findings nationwide; the inference on causality and temporal trends; and the increased risk of bias. Other limitations of this study included lack of genotypic data to identify the resistance determinants and the transmission dynamics among the human-animal-environmental interfaces. The absence of study data on the dairy farmers’ and milk consumers’ antimicrobial use practices were the other limitations of this study. Only apparently healthy human populations were included in this study, which might negatively impact the generalizability to all populations. This study considered an apparently healthy human population only, which negatively impacts the generalizability of the finding to all populations. Additionally, this study considered only one randomly selected isolate from positive samples to confirm with MALDI-TOF MS, which negatively impacts within-sample diversity and resistance heterogeneity.

## 6 Conclusion

Our study revealed a low detection rate of *E. coli* and a high prevalence of antimicrobial resistance, MDR, and MARI in milk, milking environments, and milk consumers in Addis Ababa, Ethiopia. The observed high level of antimicrobial resistance, MDR, and MARI in milk, milk consumers, and the milking environment underscores the co-occurrence of AMR *E. coli* at the dairy production interfaces. Detection of antimicrobial resistance in milk isolates to antimicrobials, which are rarely used to treat *E. coli* infections in, for instance, animal mastitis, is the greatest public health concern, as these antimicrobial agents are the last resorts available for human treatment. The findings of this study urge integrated One Health surveillance, improved hygiene, and antimicrobial stewardship.

## Data Availability

The raw data supporting the conclusions of this article will be made available by the authors, without undue reservation.
